# Dehumanization of Older Unhoused Adults: Examining Age Biases and the Limits of Empathy

**DOI:** 10.1093/geroni/igaf122.2199

**Published:** 2025-12-31

**Authors:** Brooke Dolenc Nott, Dan Dowhower, Melissa Cannon

**Affiliations:** Western Oregon University, Monmouth, Oregon, United States; Western Oregon University, Monmouth, Oregon, United States; Western Oregon University, Monmouth, Oregon, United States

## Abstract

The population of older individuals experiencing homelessness is growing in the U.S. and understanding the psychological factors influencing their treatment is crucial. Previous research highlights consistent dehumanization of unhoused individuals and the impact of ageism on the care older adults receive. However, limited work examines the compounded dehumanization faced by older unhoused adults. This study explored age-based dehumanization of unhoused individuals and whether empathy interventions could mitigate these biases. Participants (N = 399; M age = 22.5) were randomly assigned to read scenarios about unhoused adolescents or adults, with varying empathy prompts (no empathy, perspective-taking, or empathy conformity). Measures of dehumanization and helping intentions were then assessed. Results revealed older unhoused individuals faced significantly higher dehumanization than younger ones, with males and younger participants demonstrating stronger biases. Empathy interventions reduced dehumanization toward adolescents but had limited effects for older adults. However, older participants showed greater willingness to help overall. Findings suggest that intersectional biases heighten dehumanization against older unhoused individuals, potentially influencing policies and services for this population. Addressing these biases is strategic to fostering more inclusive and supportive services for all unhoused populations.

